# Challenges and predictors of exclusive breastfeeding among mothers attending the child welfare clinic at a regional hospital in Ghana: a descriptive cross-sectional study

**DOI:** 10.1186/s13006-017-0104-2

**Published:** 2017-03-09

**Authors:** Abigail Kusi-Amponsah Diji, Victoria Bam, Ernest Asante, Alberta Yemotsoo Lomotey, Samuel Yeboah, Haim Acquah Owusu

**Affiliations:** Department of Nursing, P. M. B., U. P. O., KNUST, Kumasi, Ghana

**Keywords:** Breastmilk, Infants, Mothers, Exclusive breastfeeding challenges, Exclusive breastfeeding predictors, Ghana

## Abstract

**Background:**

The challenges and predictors of exclusive breastfeeding (EBF) have been examined in many parts of the world. Considering the socio-cultural dynamics and the few research studies in Ghana, the factors that hinder and predict EBF practice in other countries may be different in the Ghanaian setting. The study therefore sought to assess the challenges and predictors of EBF among mothers attending a child welfare clinic at a regional hospital in Ghana.

**Methods:**

A descriptive cross-sectional study was carried out between January and March, 2015 to elicit information from 240 mothers who were sampled using simple random sampling technique. A validated structured questionnaire was used in collecting data on participants’ socio-demographic characteristics and reported breastfeeding practices. Participants’ breastfeeding challenges were rated on a Likert scale from 1 (not at all), 2 (mild), 3 (moderate), 4 (severe) to 5 (unbearable). In this study, EBF refers to birth of the infants up to six months.

**Results:**

The top three breastfeeding challenges of mothers were: belief that breast milk alone was not sufficient in meeting their babies’ nutritional needs [mean 3.43 (standard deviation {SD} 1.35)], short maternity leave period [mean 3.41 (SD 1.29)], and socio-cultural pressure to introduce water and artificial feeds [mean 3.39 (SD 1.28)]. Independent predictors of EBF were: infant’s age [Adjusted Odds Ratio (AOR) 0.82 (95% Confidence Interval [CI] 0.71, 0.95)], and self-employment [AOR 2.67 (95% CI 1.11, 6.41)].

**Conclusion:**

Mothers are confronted with numerous EBF challenges both at the individual and societal levels, and stakeholders need to consider these in order to support breastfeeding mothers to maximize outcomes. Reviewing the labour laws on Ghana’s maternity leave to accommodate an extended maternity leave in addition to the employee’s annual leave could further improve EBF practice rates.

**Electronic supplementary material:**

The online version of this article (doi:10.1186/s13006-017-0104-2) contains supplementary material, which is available to authorized users.

## Background

In spite of its countless benefits, many efforts to promote exclusive breastfeeding (EBF) have yielded less than desired outcomes. This may be related to the challenges mothers encounter when breastfeeding. Exclusive breastfeeding challenges occur at the maternal, infant, family, healthcare system, and at community and national level. The challenges to the practice of EBF include: cracked or sore nipples, breast engorgement [1], insufficient breastmilk production [2], disapproval and discomfort of breastfeeding in public [3], insufficient breastfeeding support from society and healthcare providers [4], short maternity leave periods [5], difficulties associated with combining breastfeeding and other maternal responsibilities [6], and emotional stress [7]. A number of variables have also been noted in the literature to predict EBF practice. Among them are infant’s age [8], maternal age, marital status [9], formal educational level [10], and occupation [11].

Studies conducted in Ghana revealed that EBF was positively associated with women’s intention to breastfeed, hospital delivery, positive attitude, living in one’s own house [12], lactation counseling [13], income and mode of delivery [10]. An analysis of the 2008 Ghana Demographic and Health Survey (GDHS) by Tampa-Naah and Kumi-Kyereme [14] also showed that, the region of birth, place of delivery and perceived birth size of baby were the independent predictors of EBF.

Similar to other African societies, the breastfeeding culture in Ghana is heavily influenced by family members, friends, society [15], and the duration of maternity leave. The Act 57 of the Ghana Labour Law also makes provisions for expectant working mothers to enjoy at least 12 weeks of paid maternity leave in addition to any accumulated annual leave [10]. Nursing mothers who have an abnormal pregnancy or multiple babies are also entitled to a possible extension of their maternity leave for a minimum duration of two weeks.

Considering the socio-cultural dynamics and the few research studies from Ghana, the factors that hinder and predict EBF practice in other countries [8, 16–18] may be different in the Ghanaian setting. The study therefore sought to examine the challenges and predictors of EBF among mothers attending child welfare clinic at a regional hospital in Ghana.

## Methods

A descriptive cross-sectional survey was conducted at the Child Welfare Clinic (CWC) of the Kumasi South Hospital, a public healthcare facility. The hospital provides secondary healthcare services and also doubles as a referral centre for other primary health facilities in the Ashanti Region and beyond. The average daily attendance at the CWC is about 60 mother-infant pairs. Some of the services provided by the clinic include growth monitoring of children, administration of childhood vaccinations, health education and counselling.

Mothers with healthy infants aged between three to nine months were eligible for inclusion in this study as maternal recall was expected to be reliable and valid in this population [19]. Mothers with infants who were preterm, twins, had birth defects were excluded from the study due to the special demands these conditions place on them.

A sample size of 240 was determined using Snedecor and Cochran’s [20] formula for cross-sectional surveys with qualitative variables. The EBF rate was estimated at 83% with a confidence interval of 95% (at a Z-score of 1.96), absolute error of precision of 5%, and a 10% non-response rate. In order to avoid selection bias, simple random sampling technique was used [21], whereby participants had to choose between two sealed opaque envelopes containing “yes” and “no”. Following informed consent, participants who chose “yes” were included in the study.

From January to March, 2015, interviews were conducted to collect participants’ data until the required sample size of 240 was obtained. Each mother was interviewed after she had received the necessary child welfare services. In all, seven hundred and forty-nine (749) women were contacted until the required sample was obtained.

Data on the participants’ demographic characteristics and EBF challenges were collected using a validated structured questionnaire. The process of validating the items in the questionnaire started with a review of the existing literature on breastfeeding challenges and predictors. Questions from the pre-tested results, which will reduce the Cronbach’s alpha value were deleted to improve internal consistency of the test items (>0.7). Cronbach’s Alpha of the 11 validated items was 0.864. Participants were asked to rate the validated challenges on a Likert scale from 1 (not at all), 2 (mild), 3 (moderate), 4 (severe) to 5 (unbearable). Unbearable meant that, the challenge was one that made the mother consider EBF cessation. The total score of EBF challenges ranged from a minimum of 11 to a maximum of 55.

Exclusive breastfeeding was defined as the practice whereby infants receive breast milk alone with no additional fluids or solids other than syrups containing vitamins, mineral supplements, or medicines. In this study, EBF refers to birth up to the time of the interview for mothers with infants up to six months of age. For those with infants aged seven to nine months, their responses were focused on their breastfeeding practice for the first six months of their child’s life [22].

Data collected were coded, entered, verified, and analysed using the Statistical Package for Social Sciences (SPSS) version 16. Participants’ socio-demographic characteristics and their EBF challenges were presented using descriptive statistics. For interval (ages of the mothers and their infants) and ordinal variables (EBF challenges) that were not normally distributed, Mann Whitney *U* test was used to determine differences between the mothers who practised EBF and those who used mixed/formula feeding. In order to determine the association of EBF practice with the categorical variables, Pearson’s chi-square test of independence or Fisher’s exact test using the Monte Carlo’s approach (when cell counts were less than 5 in a matrix greater than 2*2) was carried out. For the variables which were significantly different between mothers who practised EBF and those who employed mixed/formula feeding, binary logistic regression analysis was executed for the individual variables (unadjusted) and also adjusted for the other significant variables so as to determine the independent predictors of EBF. All tests were two-sided and *p*-value considered statistically significant at the 0.05 level.

Prior to the conduct of this study, ethical approval (Ref: CHRPE/AP/085/15) was sought from the Committee on Human Research, Publications and Ethics, Kwame Nkrumah University of Science and Technology (KNUST) following administrative approval from the Management of the Kumasi South Hospital, Kumasi.

All eligible participants were informed about the purpose and design of the study, as well as the voluntary nature of their participation. They were also assured about confidentiality and anonymity of their responses. Participants who verbally consented and picked the “Yes” option further signed a written consent form to serve as evidence for their voluntary participation.

## Results

### Exclusive breastfeeding

The EBF rate among the 240 participants was 66.7%. While 160 of the participants reported to have practised EBF, the remaining 80 participants reported to have practised mixed or formula feeding.

### Socio-demographic characteristics of participants and their Infants

The mean age of the participants was 27.72 (SD 5.13) and ranged from 19 to 39 years (Table 1). The majority of them were married (80.8%) and belonged to the Christian religion (76.7%). With regards to their educational background, few of the participants (1.7%) had no formal education. A significant number of them were employed (82.5%) while the remaining 17.5% were unemployed. The mean (SD) age of their babies was 5.79 (2.17) months, with a modal age of three months.

**Table 1:** Maternal and infant characteristics (*n* = 240)

	Total (%) *n* = 240	EBF (%) *n* = 160	Non-EBF (%) *n* = 80	*p* - value
Maternal age (years)				
18–24	78 (32.5)	46 (59.0)	32 (41.0)	0.008^a^
25–31	106 (44.2)	72 (67.9)	34 (32.1)	
32–38	52 (21.7)	40 (76.9)	12 (23.1)	
39–45	4 (1.7)	2 (50)	2 (50)	
Mean age (SD)	27.72 (5.13)	28.38 (5.16)	26.40 (4.84)	
Marital status				
Single	46 (19.2)	26 (56.5)	20 (43.5)	0.104^b^
Married	194 (80.8)	134 (69.1)	60 (30.9)	
Religion				0.131^b^
Christian	184 (76.7)	118 (64.1)	66 (35.9)	
Islamic	56 (23.3)	42 (75.0)	14 (25.0)	
Educational level				
None	4 (1.7)	0 (0.0)	4 (100.0)	0.013^c^
Basic	118 (49.2)	74 (62.7)	44 (37.3)	
Secondary	74 (30.8)	54 (73.0)	20 (27.0)	
Tertiary	44 (18.3)	32 (72.7)	12 (27.3)	
Occupation				
Unemployed	42 (17.5)	18 (42.9)	24 (57.1)	0.004^b^
Self-employed	116 (48.3)	84 (72.4)	32 (27.6)	
Privately employed	46 (19.2)	32 (69.6)	14 (30.4)	
Publicly employed	36 (15.0)	26 (72.2)	10 (27.8)	
Sex of infant				
Male	116 (48.3)	74 (63.8)	42 (36.2)	0.361^b^
Female	124 (51.7)	86 (69.4)	38 (30.6)	
Age of infant (months)				
3–6	142 (59.2)	114 (80.3)	28 (19.7)	0.000^a^
7–9	98 (40.8)	46 (46.9)	52 (53.1)	
Mean age (SD)	5.79 (2.17)			

Mann Whitney *U* test was used to examine differences between the EBF and the non-EBF group as the dependent variable (BF challenges) was ordinal and not normally distributed

Chi-square test of independence was used to determine the association between categorical variables and their statistical significance when cell counts within each cell was greater than 5

Fisher’s exact test using the Monte Carlo approach was used to determine the association between categorical variables and their statistical significance when cell counts within each cell was less than 5 for tables larger than 2*2

^a^Mann Whitney *U* test; ^b^Chi square test of independence; ^c^Fisher’s exact test using the Monte Carlo approach for tables larger than 2*2

Between mothers who practised EBF and those who used mixed/formula feeding, there were no statistically significant differences in their marital status and religion. However, mothers who practised EBF were significantly older (*p* = 0.008), had higher level of education (*p* = 0.038) and had younger infants (*p* = 0.000) relative to their non-EBF counterparts. While 30% of the mothers who used mixed/formula feeding were unemployed, only 11.2% of those who practised EBF fell in this category.

### Breastfeeding challenges

The top three breastfeeding challenges were: mothers’ perception of breastmilk alone not meeting their babies’ nutritional needs (mean 3.43 [SD 1.35]), short maternity leave period (mean 3.41 [SD 1.29]), and socio-cultural pressure to introduce water and artificial feeds (mean 3.39 [SD 1.28]) (Table 2).

**Table 2:** Breastfeeding challenges reported by participants (*n* = 240)

	Total (*n* = 240) M (SD)	EBF (*n* = 160) M (SD)	Non-EBF (*n* = 80) M (SD)	EBF Median (IQR)	Non-EBF Median (IQR)	*P* - value
Worry about breastmilk alone not meeting the nutritional needs of baby	3.43 (1.35)	3.40 (1.32)	3.50 (1.41)	4 (2–4)	4 (2–5)	0.443
Short Maternity Leave Period	3.41 (1.29)	3.44 (1.29)	3.35 (1.30)	3 (3–5)	3 (3–5)	0.744
Sociocultural pressure to introduce water and artificial feeds	3.39 (1.28)	3.34 (1.31)	3.50 (1.23)	4 (2–4)	4 (2–5)	0.367
Difficulty combining work and breastfeeding	3.18 (1.30)	3.42 (1.34)	2.68 (1.07)	4 (2–5)	3 (2–3)	*0.000*
Emotional stress	3.10 (1.19)	3.28 (1.13)	2.75 (1.23)	3 (2–4)	3 (2–4)	*0.001*
Lack of support from family, friends and society	2.74 (1.35)	2.86 (1.40)	2.50 (1.23)	2 (2–4)	2 (2–4)	0.060
Lack of support from healthcare professionals	2.66 (1.21)	2.76 (1.22)	2.45 (1.17)	3 (2–4)	2 (2–3)	*0.040*
Low breastmilk production	2.63 (1.21)	2.56 (1.16)	2.78 (1.28)	2 (2–3)	2 (2–4)	0.263
Cracked or sore nipples	2.55 (1.24)	2.49 (1.17)	2.68 (1.36)	2 (2–4)	2 (1.25–4)	0.390
Breast engorgement	2.55 (1.24)	2.49 (1.17)	2.68 (1.36)	2 (2–4)	2 (1.25–4)	0.390
Shyness when breastfeeding in public	2.05 (1.23)	2.08 (1.27)	2.00 (1.15)	2 (1–2)	2 (1–2)	0.781

Mann Whitney’s *U* test was used to examine differences in the challenges experiences by the mothers who practised EBF and those who used mixed/formula feeding, as well as their statistical significance

*M* Mean, *SD* Standard Deviation, *IQR* Interquartile Range

Apart from 3 out of the 11 validated challenges, there were no statistically significant differences in the challenges encountered by those who practised EBF and those who did not. Compared to their counterparts who practised mixed/formula feeding, those who practised EBF experienced significantly higher level challenges regarding lack of support from healthcare professionals (*U* = 5400, *p* = 0.040), difficulty combining work and breastfeeding (*U* = 4250, *p* = 0.000), and emotional stress (*U* = 4818, *p* = 0.001).

### Predictors of exclusive breastfeeding

Participants’ socio-demographic characteristics (participant’s age, baby’s age, educational level, occupation) that were significantly different in both groups of mothers were cross-tabulated with EBF practice to explore the existence of any association or otherwise between these variables.

After adjustment for all the factors that were significantly different in both groups, the independent predictors of EBF practice were: infant’s age (AOR 0.82, 95% CI: 0.71, 0.95), and self-employment (AOR 2.60, 9 5% CI: 1.09, 6.22) (Table 3). A one (1) unit increase in the monthly age of infants was associated with an 18% reduced likelihood of being exclusively breastfed (AOR 0.82, 95% CI: 0.71, 0.95). Relative to mothers who were unemployed, those who were self-employed were 2.60 times more likely to practice EBF.

**Table 3:** Logistic regression model of the significant factors associated with exclusive breastfeeding (EBF)

Variables	Unadjusted Odds Ratio (95% CI)	*p* - value	Adjusted Odds Ratio (95% CI)	*p* - value
Mother’s age (years)	1.08 (1.02, 1.15)	0.005	1.05 (0.98, 1.12)	0.194
Age of baby (months)	0.79 (0.70, 0.90)	0.000	0.82 (0.71, 0.95)	*0.007*
Educational level				
None	0.00 (0.00, 0.00)	0.999	0.00 (0.00, 0.00)	0.999
Basic	0.63 (0.30, 1.35)	0.235	0.50 (0.13, 1.92)	0.311
Secondary	1.01 (0.44, 2.34)	0.977	0.81 (.21, 3.22)	0.767
Tertiary	Reference		Reference	
Occupation				
Unemployed	Reference		Reference	
Self-employed	3.50 (1.68, 7.29)	0.001	2.60 (1.09, 6.22)	*0.032*
Privately employed	3.05 (1.27, 7.32)	0.013	2.19 (0.81, 5.89)	0.121
Publicly employed	3.47 (1.34, 8.98)	0.010	1.26 (0.27, 5.82)	0.766

## Discussion

The reported EBF rate at the time of our study among mothers from the birth of their infants up to a maximum period of six months was 66.7% and compares with studies by Tampah-Naah & Kumi-Kyereme [14], Aryeetey & Antwi [23], and Gladzah [10], in which they reported 64%, 66%, and 68% respectively. While EBF was referenced from birth to six months in Gladzah’s study [10], EBF was limited to a 24-h recall by mothers in the other studies [14, 23] Even though, the EBF rate in the current study is encouraging, it still falls below the WHO’s targeted rate of 90% [8]. This therefore, underscores the need for effective strategies to enable more women to reach the desired rate to maximize outcomes [24].

In this study, a significant association was found between EBF practice and maternal age, education, and infant’s age. Older and educated women were more likely to exclusively breastfeed their infants as reported in previous studies [11, 25]. Older and more educated women may be relatively more confident and committed to breastfeeding than their younger and less educated counterparts [26] due to the infant care experiences and education they have acquired over time [27, 28].

Common to mothers who practised EBF and those who practised mixed/formula feeding, the top three breastfeeding challenges were: breastmilk alone not meeting the nutritional needs of babies, short duration of maternity leave, and socio-cultural pressure to introduce water and artificial feeds.

The topmost challenge to EBF practice, “belief that breastmilk alone is not sufficient in meeting the nutritional needs of babies” is a well- noted barrier in the literature [29, 30]. This may be related to the inability of mothers to distinguish a “hunger cry” from other cries of the infant [29] and ignorance about the adequacy of breastmilk to provide the required nourishment [31]. The breastmilk of an adequately nourished mother provides all the nutritional energy requirements for the first six months of an infants’ life, and continues to provide up to half the energy requirements during the second half of the first year, and about one-third during the child’s second year of life [22]. This therefore calls for intensification of health education on the adequacy of breastmilk to meet the nutritional needs of infants at different stages of their life and the need to assist caregivers in differentiating between the various cries of infants.

Women employed in the public sector in the current study had higher EBF rates. In spite of this, they indicated short maternity leave period as the topmost challenge, similar to previous studies [5, 32, 33]. Increased interruption of EBF and early weaning due to nursing mothers’ resumption of work before the sixth month after delivery [34, 35] has also been reported. Extending the duration of maternity leave period [10, 36], especially in developing countries where optimal breastfeeding is a cost-effective intervention for reducing infant morbidity and mortality [5] is advocated for. Hence, reviewing the labour law on Ghana’s maternity leave to accommodate an extended maternity leave in addition to the employee’s annual leave can further improve EBF practice rates among working mothers. In contrast to several studies [18, 37, 38], being unemployed was associated with a reduced likelihood of EBF practice in this study. Decision making on EBF practice is influenced by multiple factors. Unemployed women may perceive their nutritional level as inadequate in meeting their baby’s nutritional needs [22] due to financial difficulties [39]. This, coupled with ignorance about the adequacy of breastmilk in meeting their baby’s nutritional needs might influence their decision to commence complementary feeding before six months. Changes in the Ghanaian social structure with a shift from the extended family system towards that of the nuclear family may also lead to lack of support for the nursing mother in carrying out household chores resulting in the possibility of early weaning.

Nursing mothers are often culturally pressured to give water and artificial feeds to infants [40] as reported by participants in this study as the third most constraining factor to EBF. Considering the impact society has on mothers’ initiation and duration of breastfeeding [41], public education on breastfeeding should be intensified so as to stimulate societal support for EBF practice. Midwives, public health and community health nurses can have targeted education at close relations of the pregnant woman during antenatal and postnatal periods to enhance support for EBF.

Other pressing challenges of EBF were emotional stress, lack of support from society and healthcare professionals, low breastmilk production, cracked or sore nipples, breast engorgement and shyness when breastfeeding in public. Some of these challenges also, reiterate the need for healthcare professionals to continually offer appropriate perinatal guidance and support.

Consistent with other studies conducted in Nigeria [8], Sri Lanka [42] and Iran [43], an increase in the monthly age of infants was associated with a reduced likelihood of being exclusively breastfed. This may be related to the postnatal maternity leave period and the traditional home confinement of nursing mothers which occurs in the first few months after delivery, thereby creating opportunities for EBF practice.

Social desirability bias is a potential limitation of this study as data collection occurred in a baby-friendly hospital facility, where mothers receive education on EBF. In spite of this limitation, the findings from this study contribute to our understanding of the challenges and predictors of EBF in Ghana.

## Conclusion

Mothers are confronted with numerous EBF challenges both at the individual and societal levels. The predictors of EBF should be considered by all stakeholders in order to offer appropriate support to breastfeeding mothers. Promotion of exclusive breastfeeding through education, open discussions about breastfeeding challenges and extension of maternity leave is recommended for improved outcomes.

## Additional file

Additional file 1: Dataset for this study has been attached as an additional file in SPSS format. (SAV 9 kb)

## Abbreviations

EBF: Exclusive breastfeeding
